# Acetylcholinesterase inhibitors and cognitive stimulation, combined and alone, in treating individuals with mild Alzheimer’s disease

**DOI:** 10.1007/s40520-021-01837-8

**Published:** 2021-03-24

**Authors:** Maria Devita, Fabio Masina, Daniela Mapelli, Pasquale Anselmi, Giuseppe Sergi, Alessandra Coin

**Affiliations:** 1 Department of General Psychology, University of Padua, Via Venezia 8, 35131 Padua, Italy; 2 IRCCS San Camillo Hospital, Via Alberoni 70, 30126 Venice, Italy; 3 Department of Philosophy, Sociology, Education and Applied Psychology—FISPPA, University of Padua, Via Venezia 14, 35131 Padua, Italy; 4 Geriatric Division, Department of Medicine—DIMED, University of Padua, Via Giustiniani 2, 35128 Padua, Italy

**Keywords:** AChEI treatment, Cognitive stimulation, Mild Alzheimer’s disease, Combined pharmacological and non-pharmacological treatment

## Abstract

**Backgrounds:**

Acetylcholinesterase inhibitors (AChEI) and cognitive stimulation (CS) are the standard pharmacological and non-pharmacological treatments for Alzheimer’s disease (AD).

**Aims:**

The aim of this study was to investigate the effects of these treatments, alone or combined, on the neuropsychological profiles of patients with AD.

**Methods:**

Forty participants were assigned to three groups receiving either only AChEI (*n* = 14), AChEI + CS (*n* = 15), or only CS (*n* = 11). Cognition was evaluated at baseline and after three months. Linear mixed-effects models were used to investigate differences among the treatments in terms of changes in the patients’ neuropsychological profiles.

**Results:**

Results, although preliminary because of the small sample size, suggest that a general improvement was found in patients who received AChEI + CS and those who received only CS compared with those who received only AChEI. Interestingly, individuals who received only CS showed a significant improvement in immediate memory recall than those who received only AChEI. Furthermore, the group receiving AChEI + CS showed an improvement in delayed recall than the other two groups.

**Discussion:**

The combination of AChEI and CS seems to have the greatest benefit for patients with mild AD. More interestingly, CS alone is more effective than AChEI alone, even in improving memory, considered to be the “lost” cognitive domain in AD.

## Introduction

Extensive data support the use of acetylcholinesterase inhibitors (AChEI), including donepezil, rivastigmine, and galantamine, for treating AD [1, 2]. However, a growing number of studies and an increasing awareness on the part of clinicians and researchers have started to highlight the weaknesses of pharmacological treatment alone. In fact, not only it is ineffective in some cases (a percentage of individuals, varying across the literature, are unresponsive [3]), but also it does not aim at the overall psychological being and quality of life of patients and their caregivers [4, 5]. Cognitive stimulation (CS), that consists in “engaging people living with mild to moderate dementia in a range of activity (usually in group) that are aimed at general improvement of cognitive and social functioning” is the only non-pharmacological intervention that seem to meet these needs, and that have been recommended by the National Institute for Health and Care Excellence (NICE—www.nice.org.uk/guidance/ng97) [6]. When dealing with neurodegenerative diseases, complete recovery is neither an expected nor attainable goal. However, improvements in the clinical profile (daily function, cognition, and behavioral disturbances), somatic symptoms, and quality of life are outcomes that have to some extent been obtained with pharmacological and non-pharmacological interventions [7]. While AChEI is a recognized and widely used pharmacological treatment, there is still wide variability in the implementation of non-pharmacological interventions (which are sometimes poorly structured).

The aim of the present study was to investigate the effects of AChEI and CS treatments, administered alone or combined, on the neuropsychological profiles of patients with mild AD. We hypothesized that an association of AChEI and CS would be the most effective treatment in slowing down cognitive deficiencies.

## Materials and methods

### Design and participants

A mixed within- and between-subjects repeated measures design was used. Data from 40 participants attending the Centre for Cognitive Disorders and Dementia (CDCD; University of Padua) were retrospectively, from routinely clinical practice, considered. The inclusion criteria were: a diagnosis of probable AD; a mini mental state examination (MMSE) score of 21–25/30; the ability to communicate and understand verbal and written language; the ability to physically participate in a meaningful assessment and a cognitive stimulation program. The exclusion criteria were: the presence of a learning disability; the presence of psychiatric or internal disorders, such as schizophrenia or alcoholism. After diagnosis (made by expert geriatricians, according to NINCDS–ADRDA criteria, by the comprehensive geriatric assessment, bio-humoral examination and neuro-imaging investigation, the latter to exclude other clinical diseases and secondary dementias), individuals underwent a neuropsychological assessment.

According to several, real-world conditions concerning medical, social and personal factors (i.e., “Is the patient in general good health? Can he/she be prescribed with acetylcholinesterase inhibitors? Is there any contraindication to this treatment—such as bradycardia, atrioventricular block, chronic obstructive pulmonary disease?”; “Has the patient a good social/caregiving network that allows him/her to attend cognitive stimulation sessions?”; “Does the patient want to attend the cognitive stimulation?”), patients underwent, combined or alone, pharmacological and cognitive stimulation treatments. Therefore, our sample was finally sorted as follows: only AChEI (receiving only pharmacological treatment; *n* = 14), AChEI + CS (receiving both pharmacological and non-pharmacological treatments; *n* = 15), and only CS (*n* = 11).

All participants gave informed consent to their data processing for clinical and research purposes. The study complied with the ethical standards of the 1964 Declaration of Helsinki and its later amendments or comparable ethical standards.

### Assessment/outcome measures

Cognitive profiles were evaluated at baseline and after three months of intervention as briefly described below:

- The mini mental state examination (MMSE): a well-validated screening test for identifying cognitive impairment in older adults [8]. The MMSE consists of 30 questions relating to the main cognitive areas (spatial and temporal orientation, word recording, registration, attention, calculation, recall, language, repetition, and constructive praxis). The total score ranges from a minimum of 0 to a maximum of 30.
- The brief neuropsychological examination-2 (ENB-2; [9]): a battery of 14 neuropsychological subtests: digit span, immediate and delayed recall, prose memory, interference memory (10 s), interference memory (30 s), trail making test—A, word phonemic fluency, abstraction, cognitive estimation test, intricate figures test, house figure copy, daisy drawing test, clock drawing test, and ideomotor apraxia test. An ENB-2 total score was calculated to obtain a general measure of cognitive status.

### Pharmacological treatment

Individuals were newly prescribed with AChEI (in accordance with product recommendations approved by AIFA [Italian Medicines Agency], note 85 [http://www.agenziafarmaco.gov.it/it/content/nota-85]). The choice of treatment (donepezil or rivastigmine) was left entirely to the physician’s discretion and professional judgment since no differences in the efficacy or safety of these drugs have been found [10]. According to AIFA guidelines, the standard donepezil dose is 5 mg for the first month to test the patient’s tolerance to the drug, then increased to 10 mg, at which it is maintained for the entire duration of the treatment. The standard rivastigmine dose (administered in our study by transdermal patch) is 4.6 mg for the first month, subsequently increased to 9.5 mg, and finally to 13.3 mg.

### Non-pharmacological treatment

The CS program was individually carried out and consisted in participants attending 50-min sessions twice a week over 3 months (a total of 24 sessions). To avoid potential sources of bias, the CS was administered by highly trained staff. Each cognitive stimulation session, entirely conducted in a quiet and cozy room specifically equipped with this aim at the CDCD, started with the reality orientation therapy (ROT), namely the patient’s orientation within three domains: personal, spatial, and temporal. Then, the session proceeded with structured stimulation consisting of pencil-and-paper and computerized exercises specific to each of the 5 cognitive domains assessed: memory, language, spatial and temporal orientation, attention, and logic. The pencil-and-paper exercises were taken mainly from “Dementia. 100 Exercises” (“Demenza. 100 Esercizi” [11]), while the computerized ones came from the Vienna test system—COGNIPLUS (www.schuhfried.at).

**Fig. 1:**
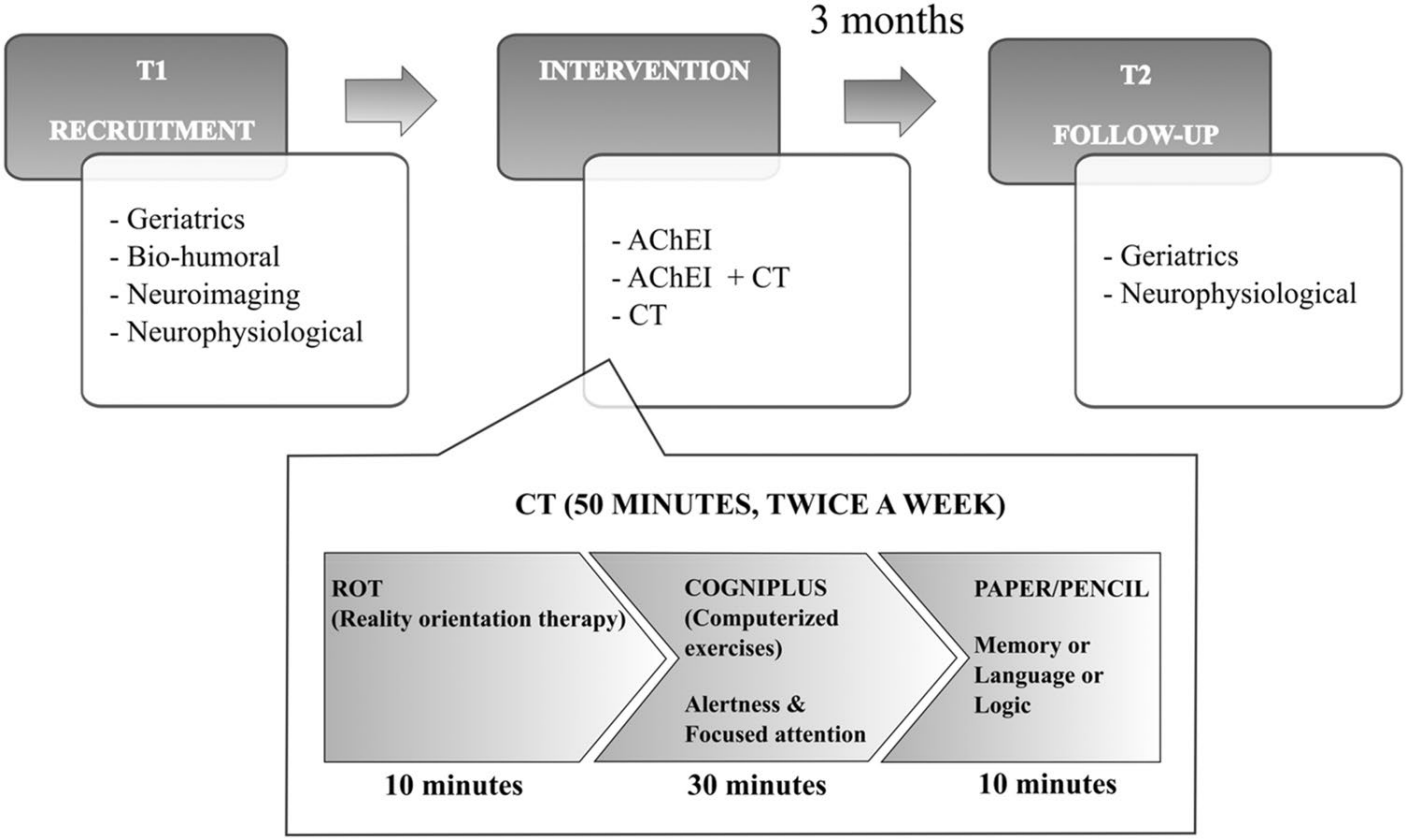
Shows the design of our study and details the activities carried out as part of the cognitive trainings

Exercises were selected for each cognitive domain and the degree of difficulty was fixed weekly, starting at a low level and becoming progressively more difficult. The same number of pencil-and-paper and computerized exercises were selected for each cognitive domain, so that all cognitive functions were equally stimulated (Fig. 1).

### Statistical analyses

The demographics (i.e., age and education) and clinical characteristics before the interventions (i.e., baseline MMSE) of the three groups were compared with ANOVAs.

Intervention effects were evaluated through linear mixed-effect models for each of the outcome measures, these being MMSE scores, ENB-2 total score, and all the ENB-2 subtests. Group (AChEI vs. AChEI + CS vs. CS) and time (T1 vs. T2) were included as fixed effects, and a random intercept was included for each subject in order to accommodate repeated measures. The significance of the fixed effects was evaluated by means of an *F* test using the Satterthwaite approximation [12]. Post hoc pairwise comparisons were corrected with the Tukey’s test. A *p* value < 0.05 was adopted for statistical significance. Statistical analyses were performed in the R environment (RStudio Team, 2020), using the R packages lme4 [13], lmerTest [14], and emmeans [15].

## Results

The ANOVAs comparing the baseline demographic and clinical characteristics of the three groups revealed no significant differences among them for age, education, and baseline MMSE scores. The descriptive statistics are shown in Table 1.

**Table 1:** Demographics and clinical characteristics of the groups before the interventions

	AChEI (*n* = 14) Males = 11, females = 3		AChEI + CS (*n* = 15) Males = 6, females = 9		CS (*n* = 11) Males = 3, females = 8		*p*
	Mean	*SD*	Mean	*SD*	Mean	*SD*	
Age	79.5	3.1	79.9	4	80.9	4	0.63
Education	9.1	4.7	10.8	5.6	9.7	5.2	0.66
MMSE (T1)	22.9	2.8	23.6	2.3	24.6	1.2	0.17

*SD* standard deviation, *CRI* cognitive reserve Index, *AChEI* acetylcholinesterase inhibitors group; *AChEI + CS* acetylcholinesterase inhibitors plus cognitive stimulation group, *CS* cognitive stimulation group

**Fig. 2:**
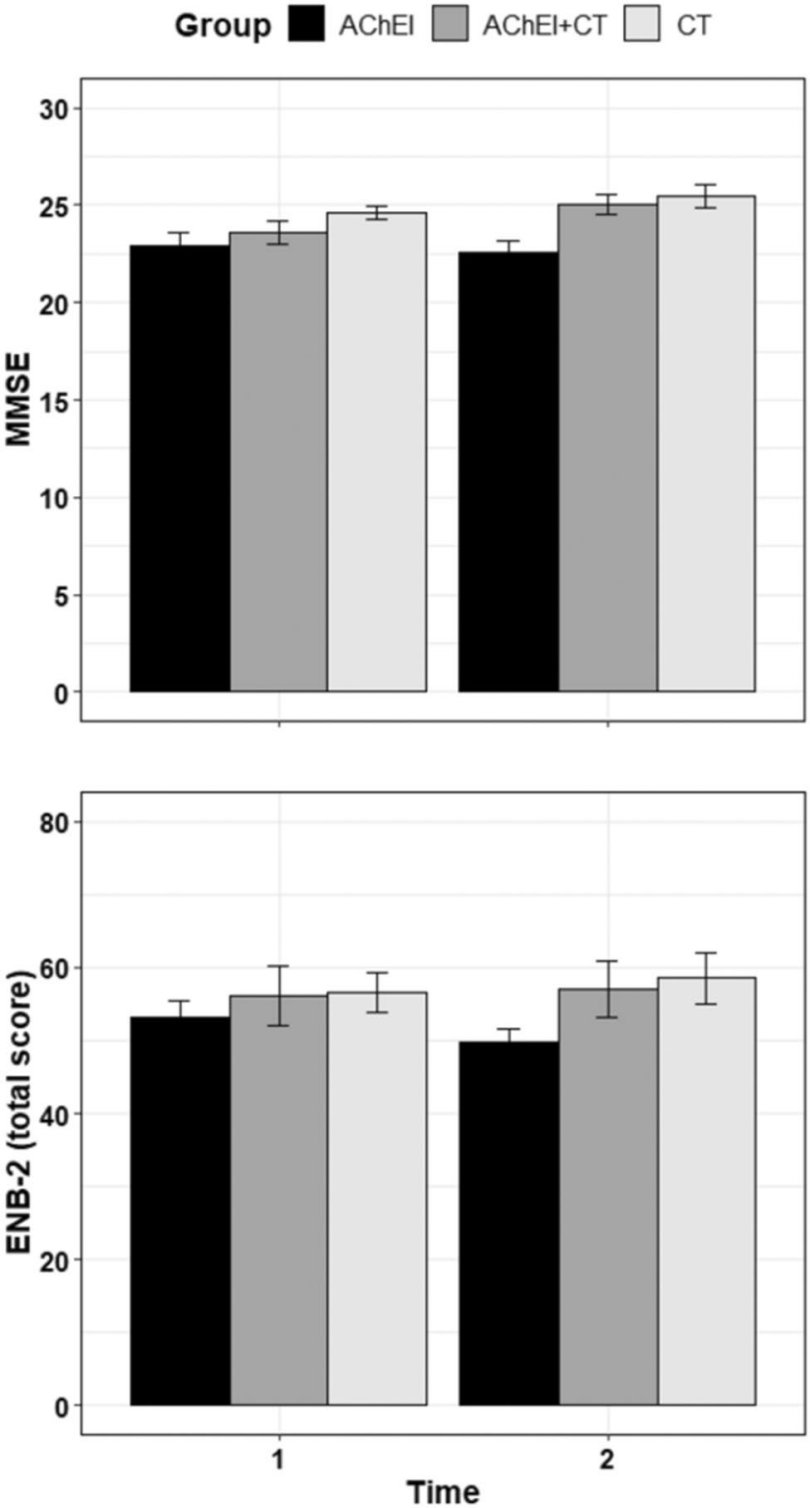
At the top, participant’s performances in the mini mental state examination (MMSE) before (T1) and after treatment (T2). At the bottom, participant’s performance in the brief neuropsychological examination-2 (ENB-2) before and after treatment

The *F* test revealed significant group differences in MMSE scores (Fig. 2) [*F*(2, 37) = 6.03, *p* = 0.005]. Post hoc contrasts showed a significant difference between the AChEI and AChEI + CS groups (22.7 vs. 24.3, respectively; *p* = 0.048), and between the AChEI and CS groups (22.7 vs. 25.1, respectively; *p* = 0.006), but no differences between the AChEI + CS and CS groups (24.3 vs. 25.1, respectively; *p* = 0.538).

A significant interaction between time and group was found for ENB-2 total scores (Fig. 2) [*F*(2, 37) = 3.55, *p* = 0.039], although post hoc comparisons showed no significant pairwise contrasts between conditions (lowest *p* = 0.216).

**Fig. 3:**
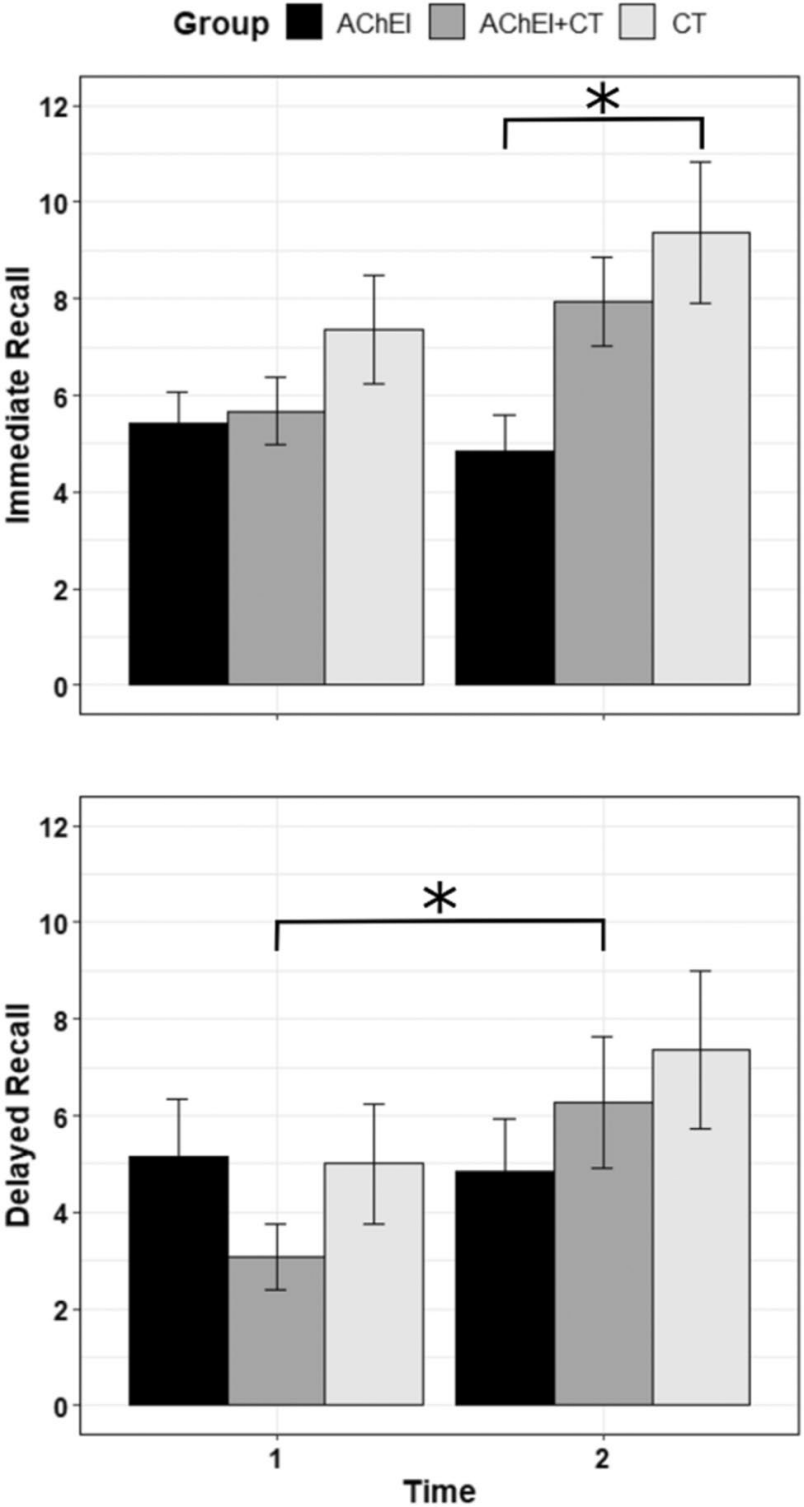
At the top, participant’s performances in the ENB-2 immediate recall prose memory subtest before (T1) and after treatment (T2). At the bottom, participant’s performances in the delayed recall prose memory subtest before and after the intervention. In both the graphs, the asterisk indicates the time × group interaction (*p* < 0.05)

With regard to the immediate recall prose memory test (Fig. 3), analyses showed a main effect of time [*F*(1, 37) = 5.62, *p* = 0.023] and group [*F*(2, 37) = 3.81, *p* = 0.031]. Scores at T2 were significantly higher than at T1 (T1 = 6.15 vs. T2 = 7.38; *p* = 0.023), and there was also a difference between the AChEI and CS groups (5.14 vs. 8.36, respectively; *p* = 0.024), although none between AChEI and AChEI + CS (5.14 vs. 6.8; *p* = 0.288) nor between AChEI + CS and CS (6.8 vs. 8.36; *p* = 0.375). In addition, the time × group interaction showed a data trend, and although the level of significance was not reached, the results suggest that there is a difference [*F*(2, 37) = 3.21, *p* = 0.052]. Post hoc comparisons revealed a significant difference between the AChEI and CS groups following treatment (4.86 vs. 9.36; *p* = 0.017).

Significant differences were found with regard to the delayed recall prose memory test (Fig. 3). *F* tests showed a main effect of time [*F*(1, 37) = 8.97, *p* = 0.005], namely higher scores at T2 than at T1 (T1 = 4.4 vs. T2 = 6.16; *p* = 0.005), and a time × group interaction [*F*(2, 37) = 3.45, *p* = 0.042]. More specifically, the AChEI + CS group showed an improvement between T1 and T2 (3.07 vs. 6.27; *p* = 0.021).

With respect to the memory with interference (30 s) test, analyses showed an interaction between time and group [*F*(2, 37) = 4.4, *p* = 0.019], although post-hoc comparisons showed no significant differences between conditions (lowest *p* = 0.232).

No significant differences emerged in the other cognitive tests: Table 2 summarizes the patient’s performance, with mean scores and standard deviations (*SD*s) of the groups at baseline (T1) and at the follow-up (T2).

**Table 2:** Patient’s performance on outcome measures. Means and standard deviations (SDs) for each group before (T1) and after (T2) treatment are shown

	AChEI				AChEI + CS				CS			
	T1		T2		T1		T2		T1		T2	
	Mean	SD	Mean	SD	Mean	SD	Mean	SD	Mean	SD	Mean	SD
MMSE	22.9	2.8	22.6	2.3	23.6	2.3	25	1.9	24.6	1.2	25.5	1.9
ENB-2 (total score)	53.1	9.1	49.7	6.8	56.1	15.8	57	14.6	56.5	8.8	58.5	11.7
Digit span	4.9	0.9	4.9	0.9	5.1	0.8	5	0.9	5.3	0.8	5.4	0.9
Immediate recall	5.4	2.3	4.9	2.8	5.7	2.7	7.9	3.5	7.4	3.7	9.4	4.9
Delayed recall	5.1	4.5	4.9	4	3.1	2.7	6.3	5.2	5	4.1	7.4	5.4
Interference memory (10 s)	4.6	2.2	3.8	1.9	4.4	3.4	4.7	3.3	5	2.6	5.5	2.7
Interference memory (30 s)	2.6	1.7	1.9	2.1	2.8	3.2	3.8	3.5	3.2	2.8	4.1	2.8
TMT-A	77.8	26.5	80.2	22.7	72.2	53.8	67.1	39.1	46.1	16.6	56.2	30.1
Fluency	9	2.6	7.9	2.6	10.7	6.3	10.9	4.9	8.5	3.1	8.2	3
Abstract reasoning	4.5	1.4	4.5	1.4	3.7	2	4.2	1.9	3.9	1.6	4.1	1.6
Cognitive estimation	3..8	0.7	3.5	1.3	3.9	1	4	0.7	4.2	1.4	4	1.2
Intricate figures	18.7	4.7	16	3.4	19.5	6.8	18.6	6.7	21	4.5	21.5	5.3
Daisy drawing	1.6	0.6	1.6	0.8	1.5	0.7	1.5	0.7	1.2	0.6	1.5	0.7
House copy	1.3	0.7	1.4	0.7	1.4	0.7	1.5	0.6	1	0.8	1.4	0.8
Clock drawing	4.5	4.7	4.1	3.8	7	3.6	5	4.2	7.8	3	6.9	3.8
Ideomotor apraxia	5.4	1.1	5.2	1.1	5.7	0.6	5.6	0.6	5.5	0.7	5.7	0.6

*SD* standard deviation, *AChEI* acetylcholinesterase inhibitors group, *AChEI + CS* acetylcholinesterase inhibitors plus cognitive stimulation group, *CS* cognitive stimulation group

## Discussion

Despite NICE guidelines and the clinician’s and researcher’s increasing awareness of the importance of cognitive stimulations in modulating the evolution of neurodegenerative disorders, there are still few resources dedicated to this non-pharmacological treatment. The evidence produced so far is often conflicting and inconsistent due to studies using different methodologies, casting doubt on the real and perceived reliability of CS. The present study aimed at comparing the efficacy of gold-standard pharmacological (AChEI) and non-pharmacological treatments (i.e., CS) for AD, administered either alone or combined, in slowing down the symptoms of cognitive decline. As shown in the results section, there were significant differences in MMSE scores between individuals receiving AChEI + CS and those receiving only AChEI, the latter performing significantly worse. Although interesting, this result was expected, since a consistent, double therapy would be predicted to be more effective than a single therapy [7, 16, 17]. More remarkable, however, was the difference in the efficacy of the two treatments administered alone: individuals undergoing only CS showed significant improvement compared with individuals receiving only AChEI. This is one of the most important findings of our study, and points toward taking a more holistic, comprehensive clinical approach that recognizes AChEI alone to be necessary but not sufficient in treating neurocognitive impairments.

Similarly, the immediate recall prose memory test showed that the CS group drew greater benefit from the intervention than the AChEI group. This result also points to the efficacy of CS, and in particular the effectiveness of recalling a short story—a simple pencil-and-paper task—as part of a cognitive stimulation session. The evidence that the CS only group performed better than the AChEI only group highlights the importance of actively stimulating memory. Also in the delayed recall prose memory test, the group receiving both treatments showed a significant improvement between the baseline and the follow-up observations. The evidence, although not definitive and certainly in need of confirmation with further studies, that memory can be improved even in individuals with neurocognitive impairments is very important and breaks new ground in the care and treatment of AD. Classic administration of CS [18] had the aim of stimulating residual cognitive functions, intentionally neglecting memory, the “lost domain”. Instead, we have shown here that it is possible—and worthwhile—to stimulate this cognitive function and thereby contribute positively to the stability of the overall cognitive profile of persons with neurodegenerative disorders and to the evolution of these diseases.

As described in the results section, our post-hoc comparisons did not always show significant pairwise contrasts between conditions, despite the significant interactions between time and group, which could be due to the small sample size. However, even in these cases, a data trend emerged, offering encouragement for further investigation.

Our findings seem to attest to the greater efficacy of CS compared with AChEI, and of AChEI combined with CS compared with single treatments, where participants have the same baseline cognitive profile. Of course, we do not even remotely suggest that AChEI be substituted with CS. However, the results show CS to be a valid and effective alternative, especially in those cases where AChEI are contraindicated (e.g., bradychardia) and where it would be too early to prescribe other medication (e.g., memantine). It is plausible that CS is more effective than AChEI alone because it offers broader, more complex stimulation than the pharmacological treatment: the individual has to get to the hospital, has a definite goal for the day, and has to meet and interact with professionals. This reasoning is made also according to the evidence that an enriched (cognitive and social) environment also has beneficial effects on neuroplasticity in older individuals [19]. The medication alone certainly has positive biochemical effects on cognition, but does not offer the individual stimulation in other areas.

### Limitations and strengths

The main limitation of the present study is certainly the sample size. We acknowledge that each group comprised only a few participants, but we feel this is justified by the significant clinical commitment that CS entails, and in light of previous studies with similar sample sizes. Another potential limitation is the lack of a fourth control group of individuals not receiving any treatment, which has, of course, ethical underpinnings. However, a possible solution to this problem could be to include individuals who, for reasons that may be clinical (contraindications to AChEI, poor compliance) or organizational (difficulty in reaching the hospital, absence of caregivers), cannot benefit from either pharmacological or non-pharmacological interventions.

Nonetheless, some strengths deserve to be acknowledged too. Our CS were particularly lengthy and intense, and it may be that previous studies did not find the same level of efficacy as ours because their treatment period was too short (2/4 weeks; [7]). Furthermore, while previous studies [7, 16] compared the effects of combined (AChEI + CS) vs. single interventions (only AChEI) in treating AD, none has included a third group receiving only CS, as we did.

In addition, the CS in our study were carefully and consistently administered by highly trained staff who were able to engage participants not only cognitively, but also emotionally and socially. Other studies that we have cited—the only ones available at the time—mainly instructed caregivers to carry out ROT at home, with no experimental control over what the individuals were actually doing. This strategy certainly has some advantages, but a possible drawback consists of an uncontrolled administration of the intervention that could compromise the efficacy of the CS. It should also be pointed out that these “informal” interventions cannot be considered as standardized CSs, with the possible consequence of leading to non-replicable results.

## Conclusions

The present study shows that a combination of pharmacological (acetylcholinesterase inhibitors) and non-pharmacological (cognitive stimulations) interventions is the ideal and most desirable treatment for AD. In addition, although further investigation is needed, long-term, structured, profoundly engaging cognitive stimulations carried out under well-controlled conditions can produce more significant improvements in cognitive functioning, and even in memory abilities, than pharmacological treatment alone. Thus, cognitive stimulations can be considered useful and efficient, even in the absence of pharmacological support.
